# Photosynthetic responses of *Larix kaempferi* and *Pinus densiflora* seedlings are affected by summer extreme heat rather than by extreme precipitation

**DOI:** 10.1038/s41598-024-56120-3

**Published:** 2024-03-04

**Authors:** Gwang-Jung Kim, Heejae Jo, Min Seok Cho, Nam Jin Noh, Seung Hyun Han, Asia Khamzina, Hyung-Sub Kim, Yowhan Son

**Affiliations:** 1 Division of Environmental Science and Ecological Engineering, Korea University, 145, Anam-ro, Seongbuk-gu, Seoul, 02841 Republic of Korea; 2 Forest Technology and Management Research Center, National Institute of Forest Science, Pocheon, 11186 Republic of Korea; 3 Research Planning and Coordination Division, National Institute of Forest Science, Seoul, 02455 Republic of Korea; 4 Department of Forest Resources, Kangwon National University, Chuncheon, 24341 Republic of Korea; 5 Institute of Life Science and Natural Resources Research, Korea University, Seoul, 02841 Republic of Korea

**Keywords:** Extreme climate, Multifactor experiment, Gas exchange, Chlorophyll, Larch, Pine, Forestry, Environmental impact, Environmental health, Ecophysiology, Forest ecology

## Abstract

The frequency and intensity of summer extreme climate events are increasing over time, and have a substantial negative effect on plants, which may be evident in their impact on photosynthesis. Here, we examined the photosynthetic responses of *Larix kaempferi* and *Pinus densiflora* seedlings to extreme heat (+ 3 °C and + 6 °C), drought, and heavy rainfall by conducting an open-field multifactor experiment. Leaf gas exchange in *L. kaempferi* showed a decreasing trend under increasing temperature, showing a reduction in the stomatal conductance, transpiration rate, and net photosynthetic rate by 135.2%, 102.3%, and 24.8%, respectively, in the + 6 °C treatment compared to those in the control. In contrast, *P. densiflora* exhibited a peak function in the stomatal conductance and transpiration rate under + 3 °C treatment. Furthermore, both species exhibited increased total chlorophyll contents under extreme heat conditions. However, extreme precipitation had no marked effect on photosynthetic activities, given the overall favorable water availability for plants. These results indicate that while extreme heat generally reduces photosynthesis by triggering stomatal closure under high vapor pressure deficit, plants employ diverse stomatal strategies in response to increasing temperature, which vary among species. Our findings contribute to the understanding of mechanisms underlying the photosynthetic responses of conifer seedlings to summer extreme climate events.

## Introduction

Extreme climate events (e.g., extreme heat, drought, and heavy rainfall), generally defined as inordinately hotter, drier, or wetter conditions compared to the historical reference period, have an increasing trend in terms of frequency, intensity, and magnitude worldwide^1–3^. For example, in Europe, the days of heatwave increased by approximately 71 in 2021 compared to those in the year of 1864, while extreme drought and rainfall increased by approximately six and three times, respectively^4^. Similarly, East Asia also experienced a rise in the frequency of such extreme climate events in recent decades^5^. Moon et al.^6^ reported that summer extreme heat and precipitation in East Asia increased by 4–8% over the past three decades. This increase has led to more frequent occurrences of extreme precipitation, culminating in events such as the 2018 Japan flood-heat wave succession^7^. Moreover, numerous studies have reported that these extreme climate events are anticipated to exhibit an increasing trend over time, across various regions^3,8–10^. For instance, if the mean temperature rises by 4.3 °C from the current level, some tropical and subtropical regions could face extreme heat for 15–20% of all days in 2100^8^. Additionally, the frequency of daily 99th percentile of precipitation in Europe is projected to increase by approximately 50% by 2100 compared to 2020^9^. Such extreme climate events can prompt changes in ecosystem functioning and damage the recovery system of plants, thereby inducing irreversible damage^2,11^.

Extreme climate events affect photosynthesis of plants. While photosynthesis typically increases with increasing temperature until an optimum point, extremely high temperature decreases the activity of photosystem II and causes damage to the thylakoid membrane, leading to a substantial reduction in net photosynthetic rate (*P*_n_)^12,13^. Furthermore, under extreme heat events, the increase in leaf-to-air vapor pressure deficit (VPD_L_) causes stomatal closure, reducing *P*_n_ and transpiration rate (*E*)^14–16^. In extreme drought, reduced soil water availability leads to stomatal closure, limiting transpiration and evaporative cooling^16–18^, thereby pushing plants towards critical temperature thresholds and decreasing chlorophyll content and *P*_n_ due to impaired chloroplast biosynthesis^19^. Although increased soil moisture from rainfall would mitigate these issues, an excessive soil moisture under heavy rainfall leads to soil saturation and flooding, and thus, reduces the availability of oxygen and nutrients to plants, resulting in stomatal closure and degradation of chlorophyll contents and *P*_n_^20,21^.

The simultaneous extreme climate events pose a greater challenge to the photosynthetic activities of plants than single events^13,16^. For example, during periods of extreme drought, plants experience thermal stresses due to limitations in their ability to cool the leaves through transpiration^22^. This situation can be aggravated with when extreme heat, intensifying the thermal stress to levels that surpass a critical threshold. These hot drought conditions can lead to functionale damage and impede the recovery of hydraulic conductance, ultimately increasing the risk of plant mortality^13,23^. It is important to note that hot and humid conditions can also amplify abiotic stresses. The combination of extreme heat and high humidity can lead to a reduction in the biochemical contents (e.g., sucrose, starch, and soluble protein) within leaves, thereby decreasing *P*_n_^24^. Furthermore, the impact of concurrent extreme climate events during summer, particularly in East Asian monsoon regions, would be exacerbated due to the highest values of both air temperature and precipitation in summer. Despite of the significant impact of combined extreme climate events on photosynthesis, there has been limited focus on them in studies^23,25^.

We selected two conifer species different in plant functional type, *Larix kaempferi* (deciduous) and *Pinus densiflora* (evergreen). Both species are commercially important and widely distributed in South Korea^26,27^. Previous studies have highlighted distinct patterns in the photosynthetic responses of these two species under environmental stress, particularly in terms of hydraulic traits. Bhusal et al.^28^ reported that a decrease in leaf water potential and *P*_n_ under water deficit conditions was observed in *L. kaempferi*, but not in *P. densiflora* seedlings in their drought experiment. However, there is lack of previous studies that specifically investigate the photosynthetic activities of these two species in response to extreme heat, either alone or in combination with drought or heavy rainfall. Understanding how extreme conditions impact the photosynthetic activities of seedlings is crucial for anticipating the potential changes in forest ecosystems’ function under the increasing frequency of extreme climate events. Therefore, this study aims to investigate the impact of summer extreme heat, drought, and heavy rainfall, as well as their interactions, on the photosynthetic activities of *L. kaempferi* and *P. densiflora* seedlings by examining leaf gas exchange parameters and chlorophyll contents. To achieve the research goals, we simulated extreme climate events in the open field in a manner that mimicked naturally occurring conditions. We formulated the following hypotheses:

1. The extreme heat treatment would decrease *g*_s_ in *L. kaempferi* and *P. densiflora* by inducing stomatal closure under high VPD_L_ (H1a), and this decrease would be most pronounced under concurrent drought treatment (H1b). Additionally, the treatments of extreme climate events would decrease chlorophyll contents (H1c).
2. As a consequence of decreased *g*_s_, *E* and *P*_n_ would decrease under the extreme heat, drought, and heavy rainfall treatments.

## Materials and methods

### Experimental design

The open-field experiment was carried out in Pocheon, South Korea (37° 45′ 38.9″ N, 127° 10′ 13.4″ E) (Fig. S1). This site is in the humid continental climate zone, characterized by hot summers and cold/dry winters^29^, with a high inter-annual variation of annual precipitation. Over a period of 23 years (1997–2019), the mean annual temperature at this site ranged from 9.2 to 11.4 °C and the annual precipitation ranged from 870 to 2329 mm.

The experimental setup consisted of six blocks, within each of which nine 1.5 m × 1.0 m plots were established (Fig. S2). Three blocks were assigned for *L. kaempferi* and the remaining three blocks were designated for *P. densiflora*. Within each plot, a random combination of two types of treatments was assigned: temperature treatments (ambient, ambient + 3 °C, and ambient + 6 °C; referred to as TC, T3, and T6, respectively) and precipitation treatments (ambient, complete exclusion of rainfall as extreme drought, and water addition above the ambient as heavy rainfall; referred to as PC, DR, and HR, respectively). Two factorial combinations were introduced, consisting of three temperature regimes and three precipitation regimes. Consequently, 54 plots were arrayed at the experimental site (two species × three temperature levels × three precipitation levels × three replicates). In April 2020, a total of 88 and 99 1-year-old *L. kaempferi* and *P. densiflora* seedlings, respectively, were planted in each plot following the guidelines for seedling management provided by Korea Forest Service (2020). The soil texture at the experimental site was classified as sandy loam (70% sand, 20% silt, and 10% clay).

Infrared heaters (FT-1000, Mor Electronic Heating Assoc., Comstock Park, MI, USA) were used to increase the canopy temperature (CT) in the T3 and T6 treatments (Fig. S4). Infrared thermometers (SI-111, Apogee Instruments, Logan, UT, USA) measured the CT of experimental plots, and dataloggers (CR1000X, Campbell Scientific, Inc., Logan, UT, USA) and relays (SDM-CD-16AC, Campbell Scientific, Logan, UT, USA) maintained the target temperature under the T3 and T6 treatments (if CT reached the target temperature, relays switched off the heaters). To monitor soil temperature (ST) and SWC, measurements were taken at a depth of 5 cm using a soil temperature/moisture sensor (SI-111, Campbell Scientific, Logan, UT, USA). An automatic rainout shelter with a transparent roof (2.0 m × 1.5 m) intercepted the natural rainfall in DR plots. The rainout shelter would close only when a rain detector (HTL-301, Haimil, Republic of Korea) detected rainfall, in order to avoid disturbance from the microclimate (e.g., light and airflow) within the plots. For the HR treatment, an artificial rainfall simulator was used. This simulator employed two spray nozzles (Unijet D5-35, Spraying Systems Co., Wheaton, IL, USA) per plot, spraying water stored in a tank. For more detailed information on the experimental design, please refer to Fig. S3 and the study conducted by Kim et al.^30^.

To determine the threshold and establish the experimental scenario of extreme climate events, we utilized meteorological data from the reference period of 1961–2019 for the months of July and August in Seoul. Since meteorological data for the research site were available only after 1997, we used the data from the nearest city, Seoul, located approximately 30 km away, as a reference (Fig. S1). The target temperatures of T3 and T6 were determined based on the difference between the mean daily maximum temperature (29.9 °C) and the 90th (33.2 °C) and 99th (36.0 °C) percentiles of the daily maximum temperature, respectively, during the reference period^1^. The duration of the extreme heat treatment was determined as the longest period of consecutive days with a daily maximum temperature above the threshold for extreme heat during the reference period, which was determined to be 7 days. For DR, it was defined as the longest period of consecutive days with daily precipitation of less than 1 mm during the reference period, which amounted to 9 days^31^. HR was defined as the 95th percentile of the daily precipitation during the reference period, which amounted to 113 mm day^−1^^32^. To determine the duration of HR, we calculated the longest consecutive period with daily precipitation exceeding the threshold of heavy rainfall, which was set at 3 days.

We simulated these extreme climate events from July–August 2020. The manipulation of temperature and precipitation was divided into two periods, with one-week of no treatment period in-between the treatment periods. The first and second DR treatments were applied on the 195–203 and 218–226 day of the year (DOY), respectively. However, during the second period, soil water content (SWC) in DR plots unexpectedly increased due to naturally occurring heavy rainfall on DOY 213–217, 219, and 222. HR was implemented on DOY 197, 200, and 203 during the first period, and on DOY 220, 223, and 226 during the second period. Temperature manipulation was carried out on DOY 204–210 and DOY 227–233.

### Measurement of photosynthetic parameters

During the experimental period of July–August 2020, in situ measurements of leaf gas exchange of *L. kaempferi* and *P. densiflora* seedlings were performed using a portable photosynthesis system (LI-6800, Li-Cor Inc., Lincoln, NE, USA) with a 3 cm × 3 cm chamber (6800-12A, Li-Cor Inc., Lincoln, NE, USA). Leaf gas exchange measurements were conducted five times throughout the experimental period. The measurements were carried out on the needles of three randomly selected seedlings per each plot. The measurements were taken at a photosynthetic photon flux density of 1000 µmol m^−2^ s^−1^, relative humidity of 50%, CO_2_ concentration of 400 µmol mol^−1^, and an ambient air temperature ranging from 27 to 33 °C. Consistent with the experimental design, gas exchange measurements were conducted between 0900 and 1500 h to minimize any diurnal variations. After field measurements were conducted, the needles were brought to the lab, where their area was determined using a scanner (Perfection V700 Photo, EPSON, Japan) and an image analysis system (WinSEEDLE, Regent Instruments Inc., Québec City, QC, Canada). This information was used to calculate the measured values of *P*_n_, *E*, *g*_s_, and the ratio of intercellular to ambient CO_2_ concentration (*C*_i_/*C*_a_) on a leaf area basis. Additionally, water use efficiency (WUE) and intrinsic water use efficiency (iWUE) were calculated using the ratios of *P*_n_/*E* and *P*_n_/*g*_s_, respectively.

To measure the chlorophyll content, the needles were cut to a length of approximately 2 mm. Subsequently, 20 ± 1 mg of cut needles were placed into vials containing 5 mL of dimethyl sulfoxide (DMSO). The vials were then incubated at 65 °C for 6 h in a water bath (HQ-DW22, Coretech Korea Co., Republic of Korea) to extract the chlorophyll. After the incubation period, the absorbance of the chlorophyll extracts was measured at 648 nm and 665 nm using a spectrophotometer (UH5300, Hitachi, Japan). The absorbance values at these wavelengths were used to calculate the chlorophyll a (Chl_a_), chlorophyll b (Chl_b_), and total chlorophyll (Chl_t_) contents using the following equations^33^:

1 $${\text{Chl}}_{{\text{a}}} = \left( {{14}.{84} \times A_{{{665}}} {-}{5}.{14 } \times A_{{{648}}} } \right) \times V \div {\text{F}}.{\text{W}}.$$

2 $${\text{Chl}}_{{\text{b}}} = \left( {{25}.{48} \times A_{{{648}}} {-}{7}.{36} \times A_{{{665}}} } \right) \times V \div {\text{F}}.{\text{W}}.$$

3 $${\text{Chl}}_{{\text{t}}} = \left( {{7}.{49} \times A_{{{665}}} {-}{2}0.{34} \times A_{{{648}}} } \right) \times V \div {\text{F}}.{\text{W}}.$$

where *A*_665_ and *A*_648_ are the absorbances at 665 nm and 648 nm, respectively. *V* is the volume of DMSO, and F.W. is the fresh weight of needles.

To calculate VPD_L_, we derived hourly air temperature and relative humidity from the automatic weather station at the experimental site. The leaf temperature for VPD_L_ calculation was obtained from the infrared thermometer in the plots. VPD_L_ was calculated based on air saturation vapor pressure (ASVP) and leaf saturation vapor pressure (LSVP) using the following equations^14,34^:

4 $${\text{ASVP}} = {610}{\text{.78}} \times {\text{e}}^{{{[T}_{{{\text{air}}}} /{(T}_{{{\text{air}}}} + {237}{\text{.3)}} \times {17}{\text{.2694]}}}}$$

5 $${\text{LSVP}} = {610}{\text{.78}} \times {\text{e}}^{{{[T}_{{{\text{leaf}}}} /{(T}_{{{\text{leaf}}}} + {237}{\text{.3)}} \times {17}{\text{.2694]}}}}$$

6 $${\text{VPD}}_{{\text{L}}} = {\text{LSVP}} - {\text{(ASVP}} \times {\text{RH}}/{100)}$$

where *T*_air_ and *T*_leaf_ are the air and leaf temperatures, respectively, and RH is relative humidity. We assumed that all plots were under the equivalent RH, considering that the experiment was conducted in an open field^35^.

### Data analysis

The effects of temperature and precipitation manipulation on the environmental factors were examined using repeated measures analysis of variance (ANOVA). Additionally, the effects of temperature and precipitation manipulation on the VPD_L_, leaf gas exchange, and chlorophyll content were determined by two-way ANOVA using a linear mixed model to account for a randomized complete block design. The block was treated as a random effect and the temperature and precipitation treatments were treated as fixed effects. The linear mixed model equation used for analysis is as follows:

7 $$Y_{ijkl} = \beta_{0} + \beta_{{1}} T_{j} + \beta_{{2}} P_{k} + \beta_{{3}} TP_{ijk} + \varepsilon_{l} + \varepsilon_{ijkl}$$

where *Y*_*ijkl*_ is the response variable in the *i*th observation (*i* = 1–5) under *j*th temperature treatment *T* (*j* = TC, T3, or T6) and *k*th precipitation treatment *P* (*k* = PC, DR, or HR) with *l*th block (*l* = 1–3). *TP* is the interaction between *T* and *P*, *β*_0_ is the intercept, *β*_*n*_ are coefficients to be estimated (*n* = 1–3), *ε*_*k*_ is the random residuals associated with the block, and *ε*_*ijkl*_ is the final residuals. As coefficients of *P* and *TP* were not statistically significant for all photosynthetic parameters (*P* > 0.05), the variables were removed from the analysis. In addition, due to an increase in SWC in the DR treatment during the second period, caused by naturally occurring heavy rainfall, we excluded the data on photosynthetic activities measured during and following this period from the analysis. We verified the statistically significant differences among precipitation treatments within temperature treatments via Tukey’s post hoc test.

The effect size for each parameter was calculated as the natural logarithm of the response ratio (RR) to compare the means of treatment (T3, T6, DR, or HR) with the means of control (TC or PC) by the following equation^36^:

8 $${\text{RR}} = {\text{ln}}({\overline{X}}_{{\text{t}}} / {\overline{X}}_{{\text{c}}} )$$

where ${\overline{X}}_{{\text{t}}}$ and ${\overline{X}}_{{\text{c}}}$ are the means of parameters in the temperature and precipitation treatment and control, respectively. The variance (*v*) of RR was calculated as:

9 $$v = \frac{{{(SD}_{{\text{t}}} {)}^{{2}} }}{{{{n}}_{{\text{t}}} {\overline{X}}_{{\text{t}}} }}{ + }\frac{{{(SD}_{{\text{c}}} {)}^{{2}} }}{{{n}_{{\text{c}}} {\overline{X}}_{{\text{c}}} }}$$

where *SD*_t_ and *SD*_c_ are the standard deviation of parameters in the treatment and control, respectively, and *n*_t_ and *n*_c_ are the sample sizes of parameters in the treatment and control, respectively. The statistical significance between the treatment and control was determined by Tukey’s post hoc test.

The relationships among VPD_L_, *E*, *C*_i_/*C*_a,_ and *P*_n_ with *g*_s_ were examined by non-linear regression. Specifically, the relationship between VPD_L_ and *g*_s_ was determined using the equation proposed by Oren et al.^37^:

10 $${g}_{{\text{s}}} = {-}{m} \times {\text{ln}}\left( {{\text{VPD}}_{{\text{L}}} } \right) + {b}$$

where *m* represents the slope and *b* represents a reference *g*_s_ at VPD_L_ = 1 kPa.

Principal component analysis (PCA) was carried out to determine the relationships among environmental factors and photosynthetic activities. All data analyses and visualizations were conducted with R version 4.2.1 at a significance level of 0.05^38^. R packages of “*lme4*” for linear mixed model^39^, “*sjstats*” for RR^40^, and “*ggplot2*” for data visualization^41^ were used.

### Research ethics

The experimental research on *L. kaempferi* and *P. densiflora* seedlings, including the collection of seedling material, complied with relevant institutional, national, and international guidelines and legislation. As the seedlings were cultivated and maintained by the National Institute of Forest Science, a joint research institute involved in this study, no specific permissions were required for the seedling collection.

## Results

### Environmental conditions

**Figure 1:**
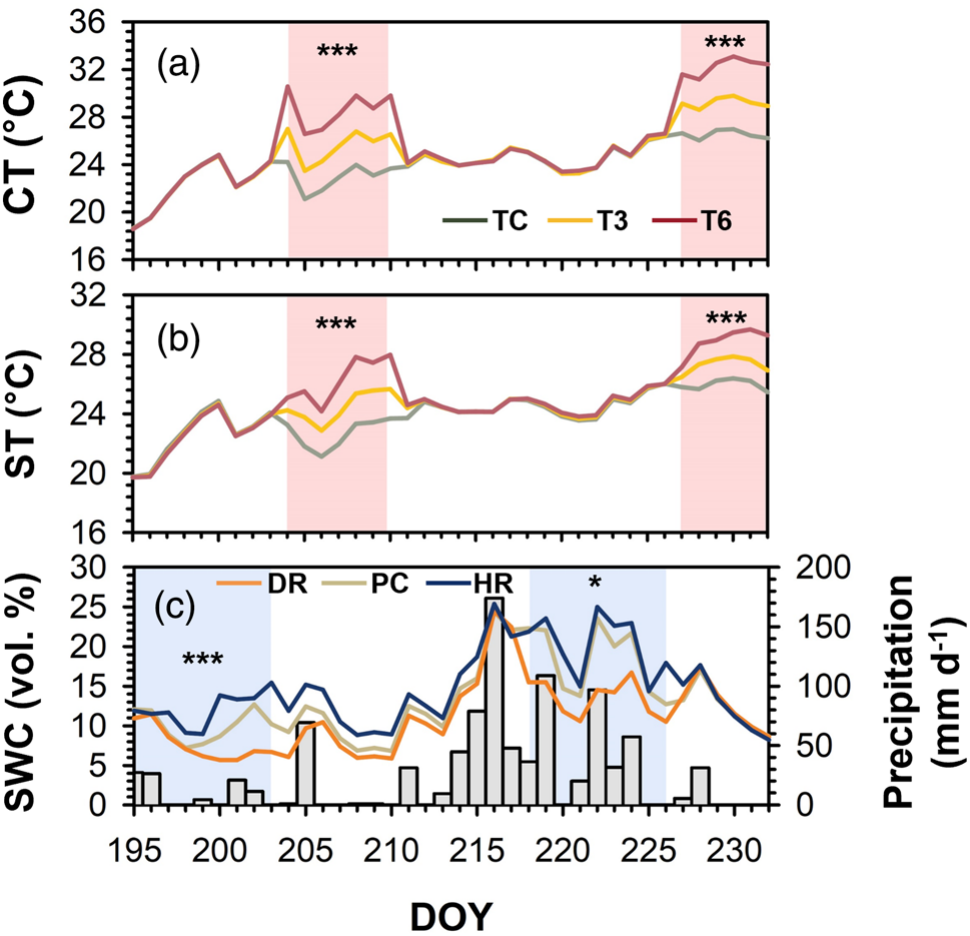
Canopy temperature (CT) (**a**), soil temperature (ST) (**b**), and soil water content (SWC) (**c**) and daily precipitation (gray bars) during the experimental period. Red and blue areas mean the period of temperature and precipitation manipulation, respectively. TC: temperature control; T3: + 3 °C treatment; T6: + 6 °C treatment; DR: extreme drought; PC: precipitation control; HR: heavy rainfall treatment. Asterisks depict statistical differences among TC, T3, and T6 in CT and ST, and DR, PC, and HR in SWC (*P < 0.05; **P < 0.01; ***P < 0.001). DOY means day of the year. This figure was modified from Kim et al.^30^.

CT during the temperature manipulation period was significantly different among TC, T3, and T6 (*P* < 0.001) (Fig. 1a). The mean CT during the first temperature manipulation period was 2.6 °C and 5.8 °C higher in T3 and T6, respectively, compared to that in TC. During the second period, the mean CT in T3 and T6 was also significantly higher (2.6 °C and 5.7 °C) than that in TC, respectively (*P* < 0.001). Temperature manipulation also significantly affected ST during both temperature manipulations (*P* < 0.001) (Fig. 1b). The mean ST (°C ± one standard error) was 22.7 ± 1.0, 24.5 ± 1.2, and 26.3 ± 1.8 in TC, T3, and T6, respectively, during the first temperature manipulation period. During the second period, ST was 26.0 ± 0.4, 27.3 ± 0.5, and 28.9 ± 0.9 in TC, T3, and T6, respectively.

There was no significant effect of temperature manipulation on SWC (*P* = 0.59) (Fig. 1c). Precipitation manipulation significantly affected SWC during the first manipulation period (*P* < 0.001). The mean SWC (vol. %) during this period was 7.8 ± 2.2, 10.1 ± 2.0, and 12.2 ± 2.1 in DR, PC, and HR, respectively. The difference in SWC among treatments during the second manipulation was also statistically significant (*P* = 0.04). The mean SWC was higher than that in the earlier manipulation, measuring 13.6 ± 2.3, 18.4 ± 4.4, and 20.3 ± 7.1 in DR, PC, and HR, respectively.

### Leaf gas exchange and chlorophyll content

**Figure 2:**
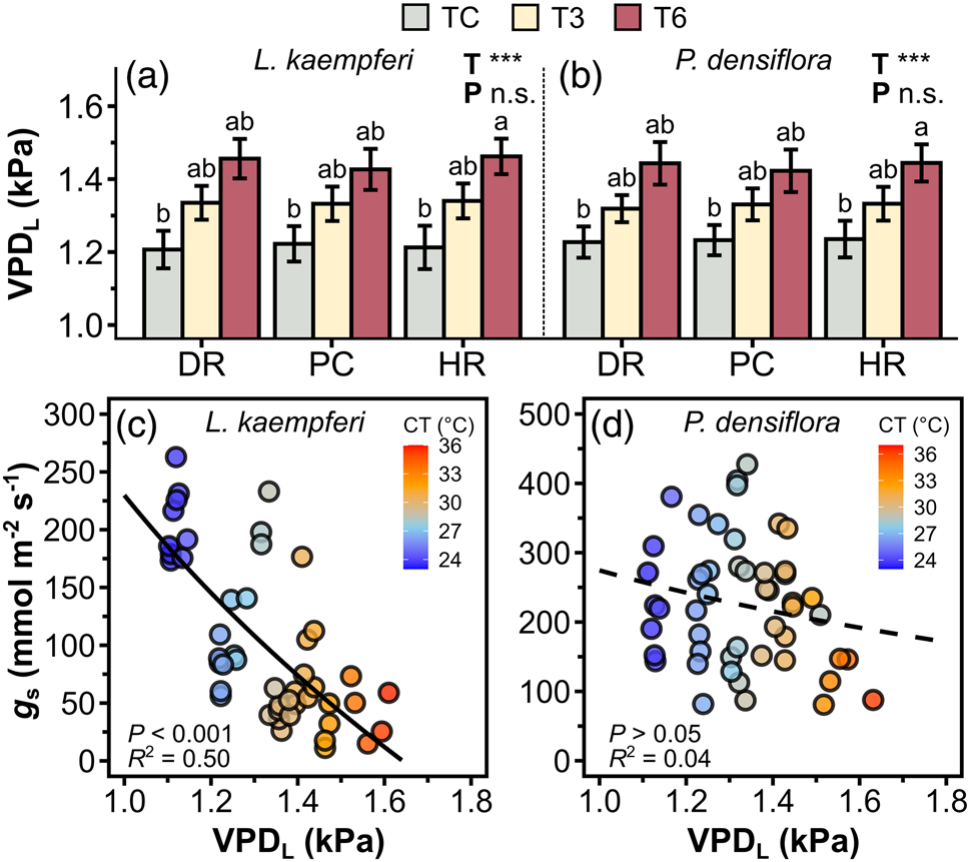
Leaf-to-air vapor pressure deficit (VPD_L_) of *Larix kaempferi* (**a**) and *Pinus densiflora* (**b**) seedlings under temperature (T) and precipitation (P) manipulation, and stomatal conductance (*g*_*s*_) as a function of VPD_L_ of *L. kaempferi* (**c**) and *P. densiflora* (**d**) seedlings. Gray, yellow, and red-colored bars indicate the temperature control (TC), + 3 °C treatment (T3), and + 6 °C treatment (T6), respectively. Colors of points are represented by canopy temperature (CT). Solid and dashed line represent the significant and non-significant regression, respectively. The regression equations are as follows: *Y* = –463.49 × ln(*X*) + 229.84 (*L. kaempferi*); *Y* = –175.73 × ln(*X*) + 274.51 (*P. densiflora*). Asterisks show the level of significance (n.s.: non-significant, *P < 0.05; **P < 0.01; ***P < 0.001). DR: extreme drought; PC: precipitation control; HR: heavy rainfall treatment.

The VPD_L_ significantly increased with increased temperature in both *L. kaempferi* and *P. densiflora* (*P* < 0.001) (Fig. 2a,b). VPD_L_ ranged between 1.10–1.67 kPa in *L. kaempferi* and 1.11–1.70 kPa in *P. densiflora*. *g*_s_ significantly decreased as VPD_L_ increased in *L. kaempferi* (*P* < 0.001) (Fig. 2c), whereas there was no significant correlation between *g*_s_ and VPD_L_ in *P. densiflora* (*P* = 0.37) (Fig. 2d).

**Figure 3:**
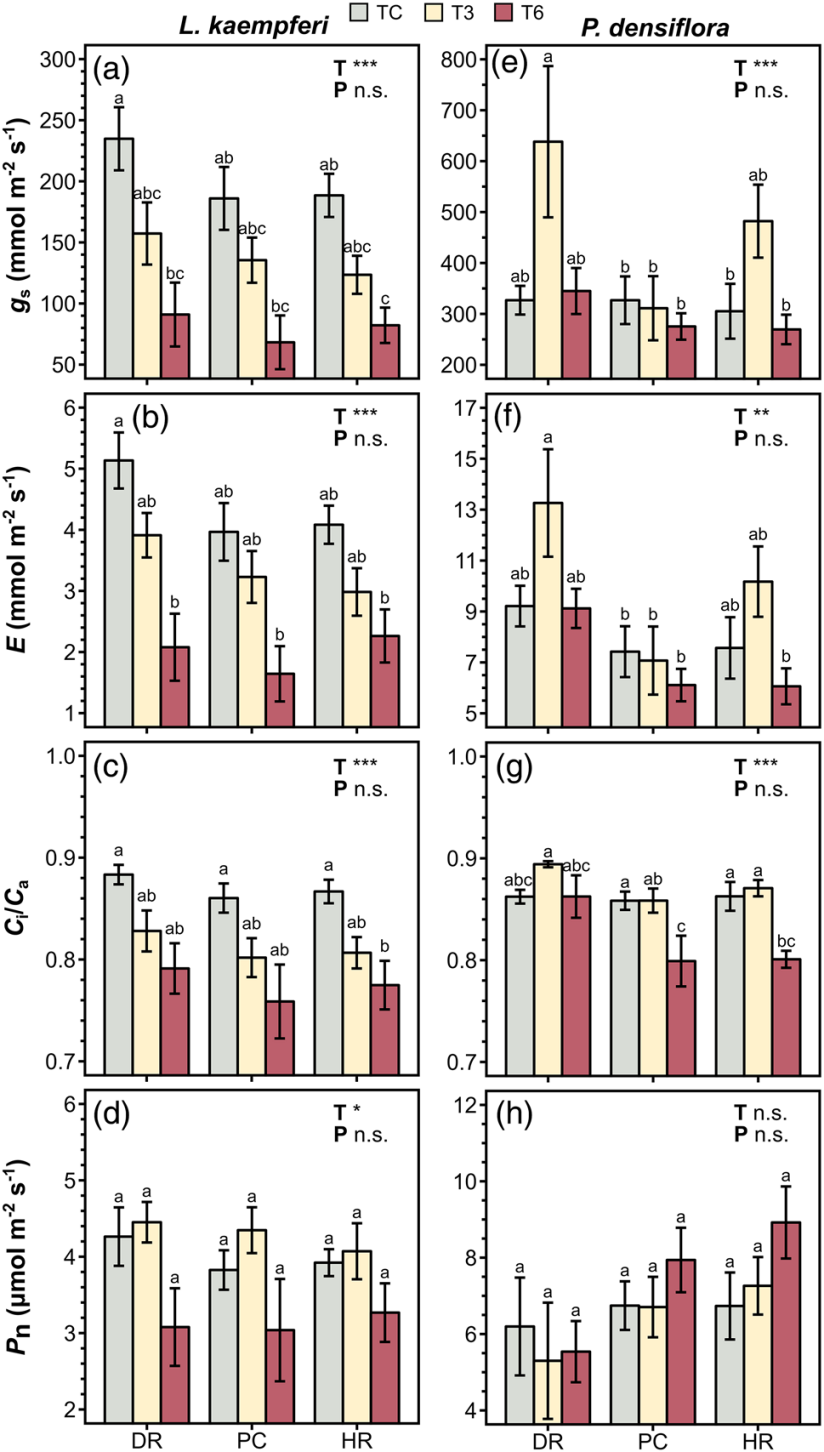
Stomatal conductance (*g*_s_), transpiration rate (*E*), ratio of intercellular to ambient CO_2_ concentration (*C*_i_/*C*_a_), and net photosynthetic rate (*P*_n_) of *Larix kaempferi* (**a**–**d**, respectively) and *Pinus densiflora* (**e**–**h**, respectively) seedlings under temperature (T) and precipitation (P) manipulation. Gray, yellow, and red-colored bars indicate the temperature control (TC), + 3 °C treatment (T3), and + 6 °C treatment (T6), respectively. Vertical lines represent one standard error of the means. Asterisks show the level of significance (n.s.: non-significant, *P < 0.05; **P < 0.01; ***P < 0.001). Different letters above the bars depict the significant differences among treatments. *DR* Extreme drought, *PC* Precipitation control, *HR* Heavy rainfall treatment.

**Figure 4:**
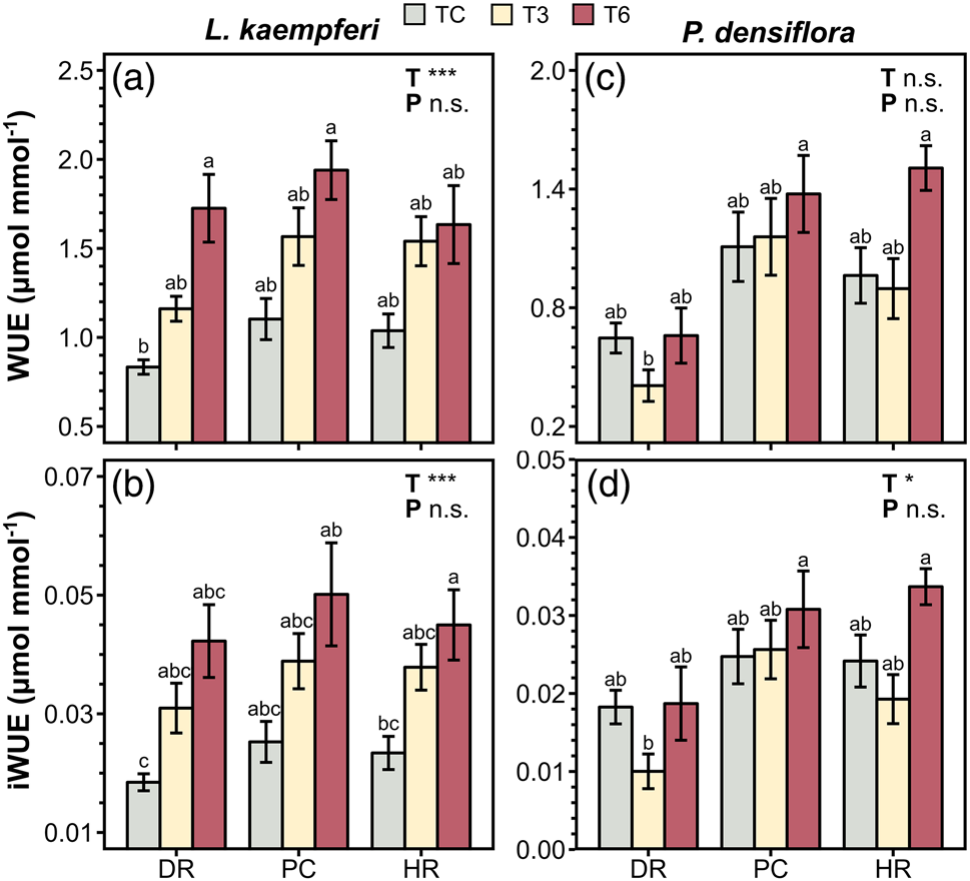
Water use efficiency (WUE) and intrinsic WUE (iWUE) of *Larix kaempferi* (**a**, **b**, respectively) and *Pinus densiflora* (**c**, **d**, respectively) seedlings under temperature (T) and precipitation (P) manipulation. Gray, yellow, and red-colored bars indicate the temperature control (TC), + 3 °C treatment (T3), and + 6 °C treatment (T6), respectively. Vertical lines represent one standard error of the means. Asterisks show the level of significance (n.s.: non-significant, *P < 0.05; **P < 0.01; ***P < 0.001). Different letters above the bars depict the significant differences among treatments. *DR* Extreme drought, *PC* Precipitation control, *HR* Heavy rainfall treatment.

**Figure 5:**
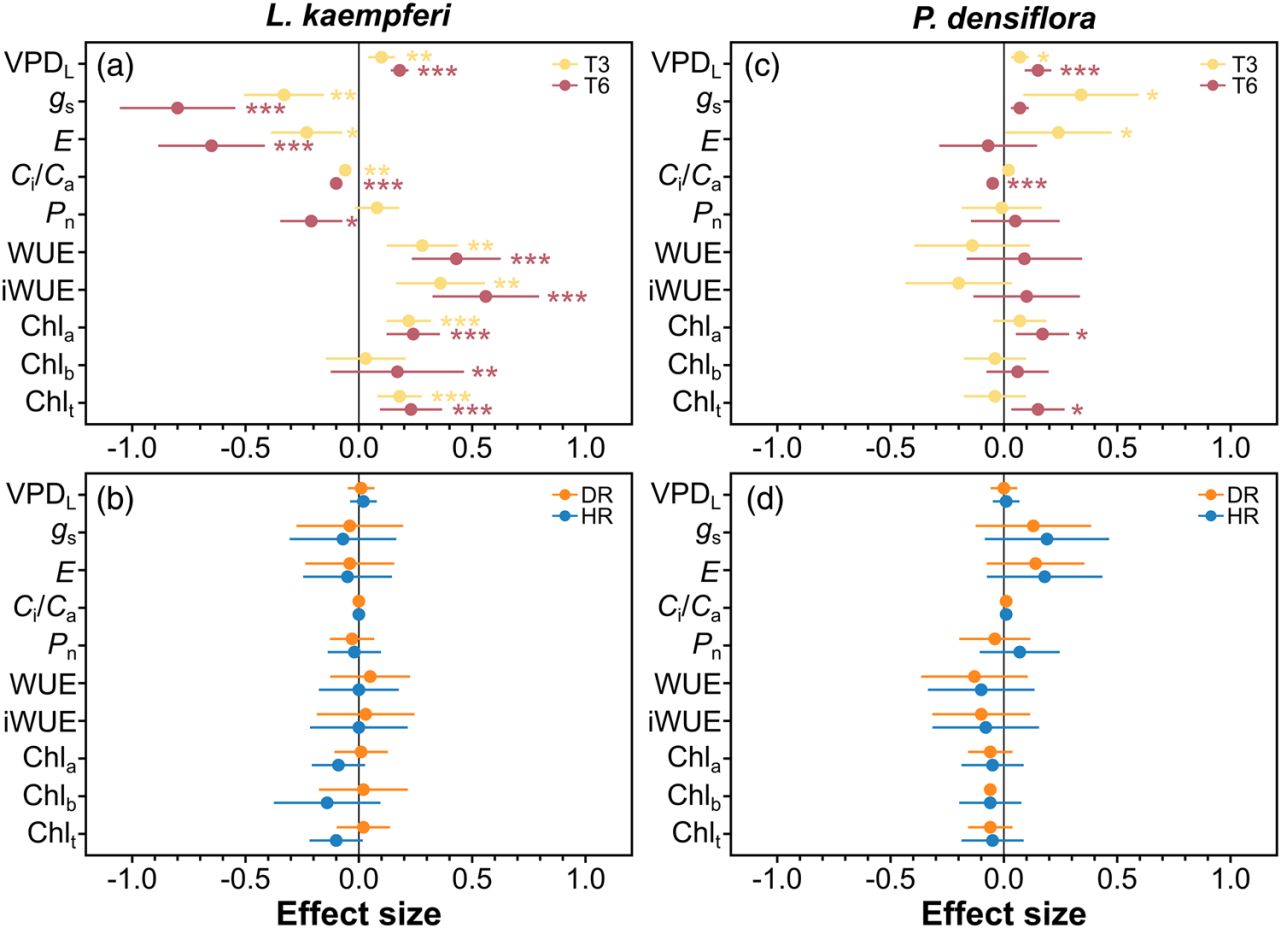
The effect size of photosynthetic parameters of *Larix kaempferi* (**a**, **b**) and *Pinus densiflora* (**c**, **d**) seedlings. Yellow and red colored-symbols indicate the + 3 °C treatment (T3) and + 6 °C treatment (T6), respectively, and orange and blue-colored symbols indicate the extreme drought (DR) and heavy rainfall (HR) treatment. Horizontal lines represent one standard error. Asterisks show the significant differences to the control (*P < 0.05; **P < 0.01; ***P < 0.001).

**Figure 6:**
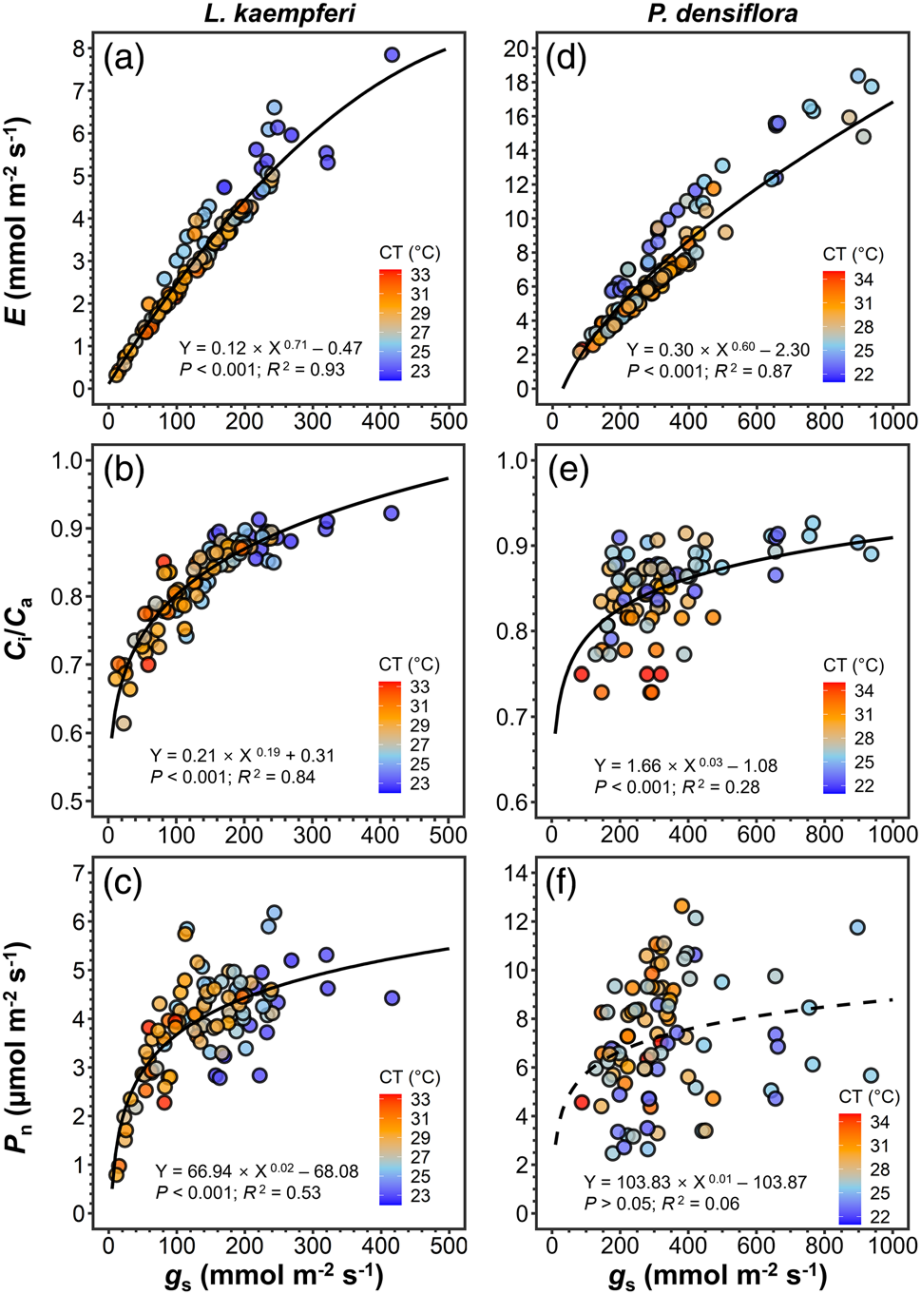
Transpiration rate (*E*), ratio of intercellular to ambient CO_2_ concentration (*C*_i_/*C*_a_), and net photosynthetic rate (*P*_n_) as a function of stomatal conductance (*g*_s_) of *Larix kaempferi* (**a**–**c**) and *Pinus densiflora* (**d**–**f**) seedlings under temperature and precipitation manipulation. Colors of points are represented by canopy temperature (CT). Solid and dashed lines represent the significant and non-significant regressions, respectively.

Temperature manipulation significantly affected *g*_s_, *E*, *C*_i_/*C*a, *P*_n_, WUE, and iWUE of *L. kaempferi* (Figs. 3a–d, 4a,b) and *g*_s_, *E*, and *C*_i_/*C*_a_ of *P. densiflora* (Fig. 3e–g), whereas precipitation manipulation and the interaction between the two treatments did not exhibit an overall effect (Figs. 3a–h, 4a–d, 5b,d; Table 1). Under T3, *g*_s_, *E*, and *C*_i_/*C*_a_ in *L. kaempferi* experienced a decrease, with effect sizes of − 0.37 ± 0.10 (*P* < 0.01), − 0.26 ± 0.10 (*P* = 0.02), and − 0.06 ± 0.01 (*P* < 0.01), respectively (Fig. 5a), whereas those in *P. densiflora* increased with effect sizes of 0.36 ± 0.15 (*P* = 0.01), 0.23 ± 0.13 (*P* = 0.07), and 0.01 ± 0.01 (*P* = 0.48), respectively (Fig. 5c). The reductions in *g*_s_, *E*, and *C*_i_/*C*_a_ in *L. kaempferi* were more pronounced under T6, with effect sizes of − 0.86 ± 0.15 (*P* < 0.001), − 0.70 ± 0.14 (*P* < 0.001), and − 0.11 ± 0.02 (*P* < 0.001), respectively, while only *C*_i_/*C*_a_ in *P. densiflora* decreased under T6 (*P* < 0.001). In addition, *E*, *C*_i_/*C*_a_, and *P*_n_ exhibited an increasing trend with increased *g*_s_ in *L. kaempferi* (*P* < 0.001) (Fig. 6a–c). However, only *E* and *C*_i_/*C*_a_ were positively correlated with *g*_s_ in *P. densiflora* (*P* < 0.001) (Fig. 6d,e), while the relationship between *P*_n_ and *g*_s_ was not statistically significant (*P* = 0.38) (Fig. 6f).

**Table 1:** Summary (*F* values) of two-way ANOVA for leaf-to-air vapor pressure deficit (VPD_L_) and photosynthetic responses of *Larix kaempferi* and *Pinus densiflora* seedlings to temperature and precipitation manipulation. Asterisks show the significant differences to the control (*P < 0.05; **P < 0.01; ***P < 0.05).

Species	Effect	*d.f*	VPD_L_	*g*_s_	*E*	*C*_i_/*C*_a_	*P*_n_	WUE	iWUE	Chl_a_	Chl_b_	Chl_t_
*Larix kaempferi*	*T*	2	14.70***	13.08***	10.69***	12.71***	3.70*	11.81***	12.35***	7.49**	0.55	5.01**
	*P*	2	0.03	0.59	0.48	0.19	0.18	0.01	0.18	1.95	0.42	1.57
	*T* × *P*	4	0.06	0.53	0.63	0.15	0.26	0.24	0.16	0.62	0.45	0.50
*Pinus densiflora*	*T*	2	13.29***	7.01**	5.58**	11.12***	0.69	2.44	3.80*	7.64**	1.80	6.62**
	*P*	2	0.03	0.08	0.13	0.32	1.06	0.24	0.31	0.22	0.17	0.22
	*T* × *P*	4	0.03	2.08	1.69	1.18	0.35	0.49	0.67	0.70	1.70	0.87

VPD_L_, Leaf-to-air vapor pressure deficit; *g*_s_, Stomatal conductance; *E*, Transpiration rate; *C*_i_*/C*_a_, Ratio of intercellular to ambient CO_2_ concentration; *P*_n_, Net photosynthetic rate; WUE, Water use efficiency; iWUE, Intrinsic water use efficiency; Chl_a_, Chlorophyll a content; Chl_b_, Chlorophyll b content; Chl_t_, Total chlorophyll content.

Chl_a_ and Chl_t_ in both *L. kaempferi* and *P. densiflora* were higher in warmer plots, whereas precipitation manipulation did not have a significant impact on chlorophyll content (Table 2). In *L. kaempferi*, the effect size on Chl_a_ and Chl_t_ in T3 was 0.26 ± 0.06 (*P* < 0.001) and 0.21 ± 0.07 (*P* < 0.001), respectively, while in T6 it was 0.26 ± 0.08 (*P* < 0.001) and 0.24 ± 0.09 (*P* < 0.001), respectively (Fig. 5a). For *P. densiflora*, the effect size on Chl_a_ and Chl_t_ were 0.08 ± 0.06 (*P* = 0.48) and − 0.04 ± 0.07 (*P* = 0.06), respectively, in T3, while it was 0.22 ± 0.07 (*P* = 0.02) and 0.20 ± 0.07 (*P* = 0.02), respectively, in T6 (Fig. 5c).

**Table 2:** Mean (SE) chlorophyll contents of *Larix kaempferi* and *Pinus densiflora* seedlings under temperature and precipitation manipulation.

Species	Manipulation		Chlorophyll contents (mg g^−1^)		
	Temperature	Precipitation	Chl_a_	Chl_b_	Chl_t_
*Larix kaempferi*	TC	DR	0.69 (0.02) ab	0.16 (0.01) a	0.85 (0.03) ab
		PC	0.66 (0.05) ab	0.17 (0.02) a	0.83 (0.08) ab
		HR	0.52 (0.03) b	0.15 (0.02) a	0.66 (0.05) b
	T3	DR	0.80 (0.05) ab	0.20 (0.04) a	1.01 (0.09) ab
		PC	0.80 (0.06) a	0.16 (0.02) a	0.96 (0.07) a
		HR	0.75 (0.05) a	0.15 (0.01) a	0.90 (0.06) ab
	T6	DR	0.77 (0.09) ab	0.17 (0.03) a	0.94 (0.12) ab
		PC	0.85 (0.15) ab	0.21 (0.02) a	1.06 (0.16) ab
		HR	0.77 (0.05) ab	0.18 (0.06) a	0.95 (0.09) ab
*Pinus densiflora*	TC	DR	0.66 (0.00) c	0.12 (0.01) b	0.78 (0.01) c
		PC	0.93 (0.04) abc	0.21 (0.01) a	1.14 (0.05) abc
		HR	0.76 (0.07) bc	0.16 (0.02) ab	0.92 (0.09) bc
	T3	DR	0.76 (0.06) bc	0.14 (0.00) ab	0.90 (0.05) bc
		PC	0.96 (0.11) abc	0.18 (0.02) ab	1.13 (0.13) abc
		HR	0.92 (0.06) abc	0.18 (0.01) ab	1.10 (0.07) abc
	T6	DR	0.82 (0.01) abc	0.17 (0.01) ab	0.98 (0.02) abc
		PC	1.10 (0.09) ab	0.20 (0.02) ab	1.30 (0.10) ab
		HR	1.15 (0.11) a	0.22 (0.02) a	1.37 (0.13) a

Chl_a_, Chlorophyll a content; Chl_b_, Chlorophyll b content; Chl_t_, Total chlorophyll content; TC, Temperature control; T3, + 3 °C Treatment; T6, + 6 °C Treatment; DR, Drought treatment; PC, Precipitation control; HR, Heavy rainfall treatment. Different letters mean the statistical significance among treatments.

**Figure 7:**
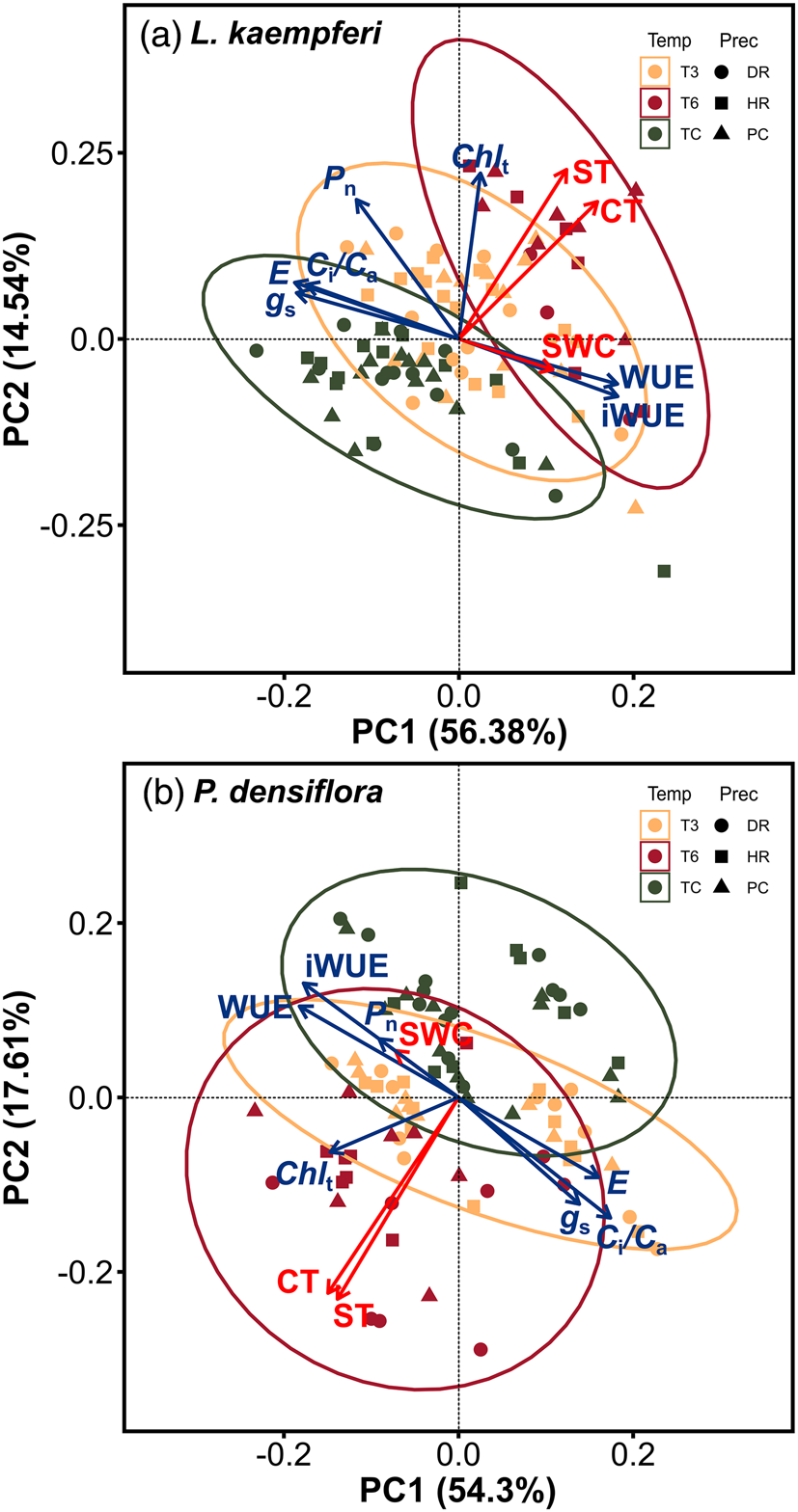
Principal component analysis (PCA) biplot for photosynthetic parameters (blue arrows) and environmental factors (red arrows) of *Larix kaempferi* (**a**) and *Pinus densiflora* (**b**) seedlings under temperature (temp) and precipitation (prec) manipulation. *TC* Temperature control; T3: + 3 °C treatment; T6: + 6 °C treatment; DR, Drought treatment; PC, Precipitation control; HR, Heavy rainfall treatment; *g*_s_, Stomatal conductance; *E*, Transpiration rate; *C*_i_*/C*_a_, Ratio of intercellular to ambient CO_2_ concentration; *P*_n_, Net photosynthetic rate; WUE, Water use efficiency; iWUE, Intrinsic water use efficiency; Chl_t_, Total chlorophyll content; CT, Canopy temperature; ST, Soil temperature; SWC, Soil water content.

By analyzing all photosynthetic parameters and environmental factors with PCA, the biplot of *L. kaempferi* showed a definite grouping by temperature treatments (Fig. 7a). PC1 of *L. kaempferi* explained 56.38% of the variations and indicated that *E*, *g*_s_, and *C*_i_/*C*_a_ were negatively related to CT, WUE, and iWUE. PC2 explained 14.54% of the total variations and indicated the positive correlation among *P*_n_, Chl_t_, CT, and ST. The results of PCA for *P. densiflora* seedlings showed that PC1 and PC2 explained 54.30% and 17.61% of the total variations, respectively (Fig. 7b). PC1 revealed that *E* and *C*_i_/*C*_a_ were negatively related to the WUE and iWUE of *P. densiflora* and PC2 explained the relationship among environmental factors.

## Discussion

The functionality of temperature and precipitation manipulation systems is crucial when conducting experiments in the open field, as ambient climate factors can easily influence the experimental treatments^2^. In this study, the temperature manipulation system successfully simulated the conditions of real extreme heat events. The temperature manipulation system ensured that the heated and ambient plots had distinct CT and ST conditions, even during periods of rainfall (DOY 205 and 228) or under extremely high air temperature (33.1 °C on DOY 232; data not shown) conditions. However, there was an unexpected increase in SWC in DR plots during the second manipulation period. These results were inconsistent with those that would occur under water stress, and we propose that the cause of the increased SWC was the naturally occurring extreme precipitation events. Specifically, during the rest period between the two periods of experimental precipitation manipulation, the study area received a significant amount of rainfall (358 mm) over a five-day period (DOY 213–217), which accounted for 26% of the mean annual precipitation over 23 years. It is suggested that the rainfall may have entered the plots through open sides, resulting in an increase in SWC in DR plots. These unexpected results emphasize a limitation commonly associated with the open-field experiment. Thus, we suggest considering the edge part of the plots as a buffer zone to minimize the potential influence of ambient factors, such as lateral influx of rainfall or microclimate variations when conducting the open-field experiment.

Consistent with our hypothesis (H1a), as the temperatures increased, *g*_s_ in *L. kaempferi* showed a decreasing trend (Fig. 3a). This result is consistent with the concept that high temperatures can lead to stomatal closure in order to mitigate water loss^14,42^. Elevated evaporative demand and subsequent leaf water loss associated with high temperatures may also contribute to stomatal closure^18,43^. The significant negative relationship between *g*_s_ and VPD_L_ in *L. kaempferi* found in this study was supported by these established theories (Fig. 2c). This decreasing trend in *g*_s_ under high temperatures and VPD_L_ confirms the findings of a previous study by Ameye et al.^44^ wherein the photosynthetic responses of *P. taeda* seedlings were examined under experimental heat waves with a biweekly + 6 °C treatment. The closure of stomata is likely responsible for the reduction in *E* and *C*_i_/*C*_a_^14,15,45,46^. In our study, the positive relations observed between *E*, *C*_i_/*C*_a_, and *g*_s_ in *L. kaempferi* as well as PCA results provide further evidence that the decrease in *E* and *C*_i_/*C*_a_ is associated with stomatal closure, thus in line with H2. The decrease in CO_2_ uptake can lead to oxidative damage and a decline in *P*_n_^47,48^. Additionally, heat stress may inhibit the CO_2_ fixation of plants and damage components of their photosynthetic apparatus, especially photosystem II, which plays a crucial role in electron transport during photosynthesis, as leaf temperature increases^47^. However, in our study, *P*_n_ of *L. kaempferi* significantly decreased only under T6, but not under T3. This result suggests that the relatively lower VPD_L_ under T3, compared to T6, was not sufficient to inhibit *P*_n_, since the sensitivity of *P*_n_ to VPD_L_ is weaker than that of *g*_s_^14^.

In contrast to *L. kaempferi*, the increasing VPD_L_ did not have a significant impact on *g*_s_ of *P. densiflora* (Fig. 2d). Furthermore, *g*_s_ and *E* of *P. densiflora* increased under T3 and decreased again in T6 (Fig. 5c). These results are contrary to H1a, suggesting species-specific variation of stomatal behaviors in responses to temperature and VPD_L_. The observed peak responses of *g*_s_ and *E* to increasing VPD_L_ and temperature can be interpreted as a trade-off between the ‘feed-back’ and ‘feed-forward’ stomatal responses. Stomatal transpiration can increase as a strategy for cooling the leaf surface under high temperatures, representing a feed-back response^49^. Conversely, a feed-forward response involves a decline in *E* as temperature and VPD_L_ increase to avoid hydraulic failure^43,50^. During the feed-back response, the evaporative cooling strategy may induce water loss through the leaf cuticle. Subsequently, *g*_s_ and *E* may begin to decrease after reaching a certain level of VPD_L_ and temperature in response to this water loss, aiming to prevent hydraulic failure, thus exhibiting a peak function^14,51,52^. Although these peaked responses have been a subject of debate as it is difficult to explain the response from simple stomatal mechanisms, previous studies have suggested that there is an optimum VPD_L_ and temperature for *E*^51^. Furthermore, previous findings have observed that these responses are more likely to occur when temperature co-varies with VPD_L_, rather than when temperature remains constant. The observed peaked response of *g*_s_ and *E* of *P. densiflora* under the extreme heat treatment can be interpreted as a result of the trade-off between the evaporative cooling and minimizing water loss, particularly given the covariation of temperature and VPD_L_ in our experiment.

These divergent stomatal strategies in response to extreme heat may be attributed to differences in the species’ hydraulic traits^14^. Specifically, the high sensitivity of *g*_s_ to VPD_L_ in *L. kaempferi* suggests an isohydric behavior. In contrast, *P. densiflora* appeared to withstand the extreme heat stress through anisohydric stomatal regulation, as evidenced by its consistent *P*_n_ under the treatments. In addition, the distinction in needle morphology could contribute to variation in stomatal behaviors between the two species. Longer needles are likely to receive a higher irradiance on their surface, resulting high requirement for CO_2_ uptake, compared to shorter needles^53,54^. This demand is met through increased leaf hydraulic conductance, *g*_s_, and evaporative demand. Furthermore, longer needles possess a higher hydraulic capacity, essential for delivering the water needed to maintain open stomata^53^. These hydraulic traits of a long leaf are evidenced by higher values of *g*_s_, *E*, and *P*_n_, and feed-back stomatal response in *P. densiflora* in this study which has a longer needle (approximately 5.93 ± 0.23 cm needle^−1^) compared to *L. kaempferi* (approximately 2.31 ± 0.05 cm needle^−1^). Additionally, the evergreen characteristics of *P. densiflora* likely contribute to the differential stomatal behavior, as evergreen species may invest more in carbon uptake due to their longer leaf lifespan, in contrast to deciduous species such as *L. kaempferi*, which have ‘disposable’ leaves^55^. The distinct hydraulic regulations might be a critical factor in plant mortality under environmental stresses^56^. Anisohydric behavior may provide short-term tolerance to heat stresses, as observed in our study. However, the prolonged hot and dry conditions can rapidly lead anisohydric species to dehydration and xylem cavitation, ultimately resulting in mortality due to their increased *E* at high temperatures^16^. Therefore, examining photosynthetic activities under long-term environmental stresses is essential for predicting plant survival following extreme climate events.

Interestingly, *P*_n_ in *P. densiflora* did not show changes with increasing temperature and there was no observed correlation between *P*_n_ and *g*_s_ (Figs. 3h, 6f). These findings suggest that *P*_n_ in *P. densiflora* may be influenced by factors other than *g*_s_, indicating a more complex relationship between stomatal behavior and photosynthetic performance. A study by Urban et al.^22^ examining gas exchange variables in responses to increasing temperature in *P. taeda* and *Populus deltoides* x *nigra* also observed the decoupled relationship between *g*_s_ and *P*_n_ at leaf temperatures > 40 °C, further supporting the complex interactions between these variables. In addition, a peak response of gs to increasing VPD_L_ was likely a contributing factor to the decoupling between *g*_s_ and *P*_n_^57^. These results highlight the need for further research to explore underlying mechanisms influencing *P*_n_ in *P. densiflora* and to elucidate the factors that contribute to its photosynthetic response under extreme temperature conditions.

Contrary to our hypothesis (H1c), our study found markedly high chlorophyll contents under the extreme heat treatment for both *L. kaempferi* and *P. densiflora*. While chlorophyll contents generally tend to decrease under thermal stress due to leaf senescence, there may exist an optimal temperature range where chlorophyll contents can increase as temperature rises. Previous studies have found that high temperatures may enhance plant growth and accelerate pigment biosynthesis, and the activity of enzymes involved in chlorophyll production, resulting in an increase in chlorophyll contents^58,59^. However, upon examining the growth and biomass data from the current study (data not shown), the temperature treatments did not have an impact on seedling growth and biomass in either species. Seedling height (cm) and total biomass (g seedling^−1^) of *L. kaempferi* and *P. densiflora* were not affected by the temperature treatment (*P* = 0.27 and 0.88, respectively, for *L. kaempferi*; Noh et al.^26^, and *P* = 0.61 and 0.85, respectively, for *P. densiflora*; unpublished data), ranging from 48.6–57.5 and 13.24–15.88, respectively, in *L. kaempferi*, and from 28.6–29.7 and 6.89–7.96, respectively, in *P. densiflora*. Consequently, the increased chlorophyll contents in the extreme heat treatment might be attributed to the accelerated pigment biosynthesis rather than the enhanced growth of seedlings. Yun et al.^27^ also observed that Chl_t_ in *P. densiflora* increased within the air temperature range of 15–31 °C in their experiment, where an increase in air temperature by 3 °C was simulated using the infrared heater. In our study, the observed high chlorophyll contents under extreme heat, with CT ranging from 24 to 32 °C, suggest that this temperature range may correspond to the optimum conditions for pigment biosynthesis.

In general, water deficit conditions typically lead to stomatal closure and a reduction in CO_2_ uptake, thereby reducing *g*_s_, *E*, and *P*_n_^60,61^. However, in this study, we did not observe a decrease in photosynthetic parameters under DR in both *L. kaempferi* and *P. densiflora* (Fig. 5b,d). This result could be attributed to the brief duration of summer drought spells experienced in the East Asian monsoon climate of the study region. While the lack of rainfall per se can be considered “extreme”, the brief nature of these drought spells may not have been sufficient to evoke significant changes in the photosynthetic activities. In addition, the naturally occurring extreme rainfall during the rest period likely counteracted the effect of water treatments by considerably raising the water availability level throughout the site. These findings highlight the importance of considering the dynamic nature of rainfall patterns in the East Asian monsoon regions, as summer rainfall events can provide adequate SWC for sustaining photosynthetic activities, mitigating the negative effects of summer extreme drought conditions.

Consequently, although the SWC in HR was significantly higher than that in DR and PC, the difference did not translate into photosynthetic responses to water treatments. The natural rainfall during the rest period and the second manipulation period seemed to have already produced enough SWC in PC, which met the water demand for photosynthesis, regardless of HR treatment. In a study investigating the mechanisms linking increased rainfall and water dynamics, Lopez et al.^62^ found that the crown *E* of *L. cajanderi* in an irrigated plot (under SWC of 25–28 vol.%) did not differ substantially from that in the ambient plot (under SWC of 15–25 vol.%). Similarly, Jo et al.^63^ did not observe notable differences in the *P*_n_, *E*, and *g*_s_ of *Abies holophylla* and *A. koreana* when the SWC increased from approximately 15 to 25 vol. %. Moreover, due to the soil texture (sandy loam) in this study, which has a relatively low water holding capacity^64^, it is unlikely that the difference in SWC between HR and PC plots persisted for a long period of time after the HR treatment.

## Conclusion

To summarize, we found that *L. kaempferi* showed a decrease in *g*_s_ under extreme heat, leading to the reduction in all photosynthetic parameters, whereas *P. densiflora* showed a peak function in *g*_s_ and *E* under extreme heat and no change in *P*_n_. No effect was observed of extreme drought and heavy rainfall on photosynthetic activities in both species. These findings reveal the species differences in stomatal behaviors in response to increasing temperature and *L. kaempferi* experiencing more pronounced adverse effects on photosynthesis compared to *P. densiflora*. These results indicate that extreme heat may have a more negative impact on forest succession dynamics and degrade ecosystems’ function in newly established *L. kaempferi* forests, by inhibiting carbon uptake, in comparison to *P. densiflora* forests. Therefore, these findings suggest the significance of implementing temperature management strategies in nursery systems, particularly for *L. kaempferi*, to effectively respond to extreme climate events. However, we note that the observed responses spanned only a single season, whereas experimental treatments in open-field trials can be inferred by natural environmental stochasticity, necessitating a long-term study. Thus, further long-term studies are needed to assess the lagged effect of summer extreme conditions in subsequent seasons and the recovery capacity of plants from extreme climate events.

### Supplementary Information

Supplementary Information.

## Data Availability

The data that support the findings of this study can be obtained from the corresponding author upon reasonable request.
