# Supplementary material for: Photosynthetic responses of Larix kaempferi and Pinus densiflora seedlings are affected by summer extreme heat rather than by extreme precipitation
Source: Sci Rep. 2024 Mar 4;14:5250. doi: 10.1038/s41598-024-56120-3 (PMC10912299; doi:10.1038/s41598-024-56120-3)
Supplement: Supplementary file 1 — Supplementary Information. [file 41598_2024_56120_MOESM1_ESM.docx]

**Photosynthetic responses of *Larix kaempferi* and *Pinus densiflora* seedlings are affected by summer extreme heat rather than by extreme precipitation**

Gwang-Jung Kim^1^, Heejae Jo^1^, Min Seok Cho^2,3^, Nam Jin Noh^4^, Seung Hyun Han^2^, Asia Khamzina^1^, Hyung-Sub Kim^1,5^, and Yowhan Son^1,*^

^1^ Division of Environmental Science and Ecological Engineering, Korea University, Seoul 02841, Republic of Korea

^2^ Forest Technology and Management Research Center, National Institute of Forest Science, Pocheon 11186, Republic of Korea

^3^ Research Planning and Coordination Division, National Institute of Forest Science, Seoul 02455, Republic of Korea

^4^ Department of Forest Resources, Kangwon National University, Chuncheon 24341, Republic of Korea

^5^ Institute of Life Science and Natural Resources Research, Korea University, Seoul 02841, Republic of Korea

*** Corresponding author: Yowhan Son**

Tel. +82-2-3290-3015

Fax. +82-2-3290-3651

E-mail: [yson@korea.ac.kr](mailto:yson@korea.ac.kr)

Address: Division of Environmental Science and Ecological Engineering, Korea University, 145, Anam-ro, Seongbuk-gu, Seoul 02841, Republic of Korea

**
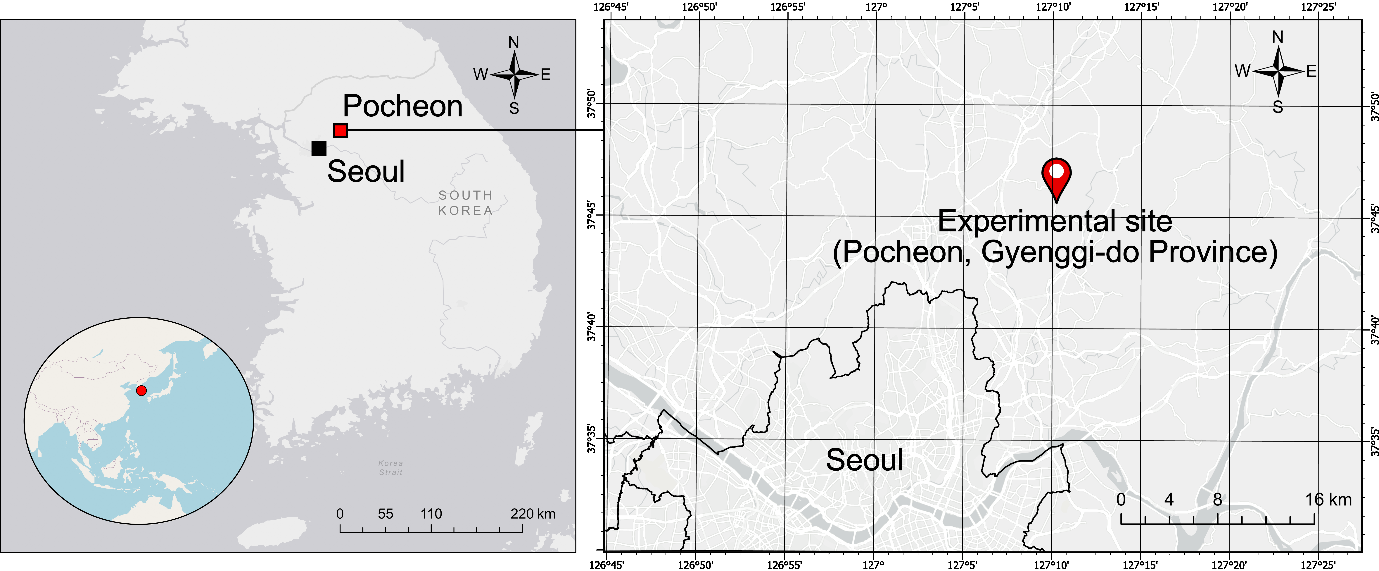
**

**Figure S1**. Location of experimental site in this study. The map was produced using ArcGIS Pro v2.6.0 (Esri, Redlands, CA, USA; https://www.esri.com).


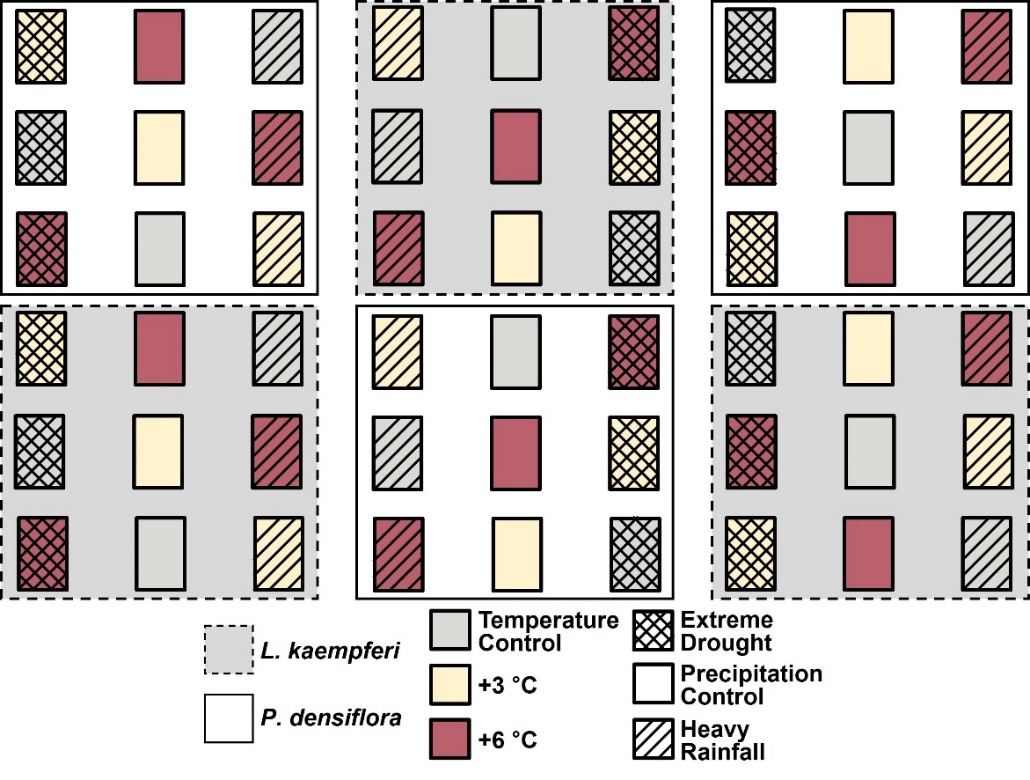


**Figure S2**. Experimental design in the current study. Gray- and white-colored blocks indicate plots covered with *Larix kaempferi* and *Pinus densiflora* seedlings, respectively. Green, yellow, and red mean temperature control (TC), +3 °C treatment (T3), and +6 °C treatment (T6), respectively. Crosshatched, none and diagonal lined patterns are extreme drought (DR), precipitation control (PC), and heavy rainfall (HR), respectively.


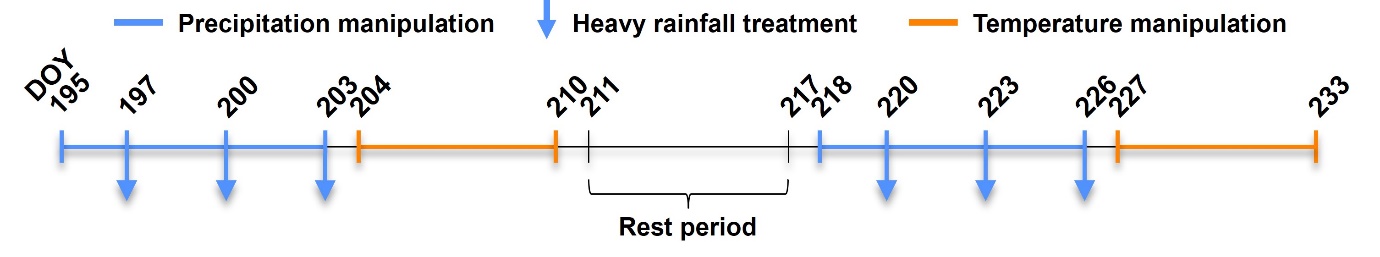


**Figure S3**. Experimental scheme of this study. Blue- and orange-colored lines show the period for precipitation and temperature manipulation, respectively. Blue-colored arrows are the dates of heavy rainfall treatments.


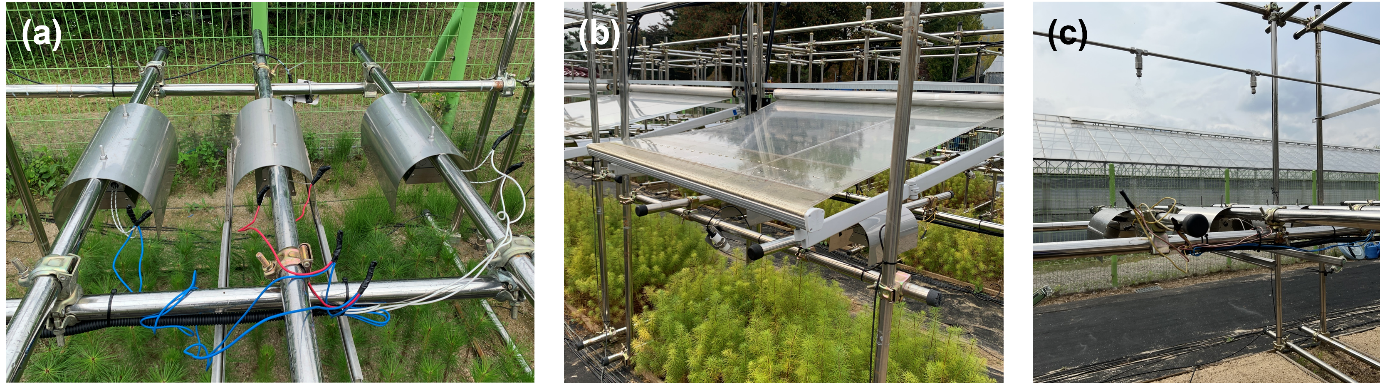


**Figure S4**. Temperature and precipitation manipulation systems using (a) infrared heaters, (b) automatic rainout shelter, and (c) spraying nozzles.
